# Dietary Protein Requirements in Children: Methods for Consideration

**DOI:** 10.3390/nu13051554

**Published:** 2021-05-05

**Authors:** Joshua L. Hudson, Jamie I. Baum, Eva C. Diaz, Elisabet Børsheim

**Affiliations:** 1 Department of Pediatrics, University of Arkansas for Medical Sciences, Little Rock, AR 72205, USA; ECDiazfuentes@uams.edu (E.C.D.); EBorsheim@uams.edu (E.B.); 2 Arkansas Children’s Nutrition Center, Little Rock, AR 72202, USA; 3 Arkansas Children’s Research Institute, Little Rock, AR 72202, USA; 4 Center for Human Nutrition, Department of Food Science, University of Arkansas System Division of Agriculture, Fayetteville, AR 72704, USA; baum@uark.edu; 5 Reynolds Institute on Aging, Department of Geriatrics, University of Arkansas for Medical Sciences, Little Rock, AR 72205, USA

**Keywords:** adolescents, muscle, fitness, amino acids

## Abstract

The current protein requirement estimates in children were largely determined from studies using the nitrogen balance technique, which has been criticized for potentially underestimating protein needs. Indeed, recent advances in stable isotope techniques suggests protein requirement as much as 60% higher than current recommendations. Furthermore, there is not a separate recommendation for children who engage in higher levels of physical activity. The current evidence suggests that physical activity increases protein requirements to support accretion of lean body masses from adaptations to exercise. The indicator amino acid oxidation and the ^15^N-end product methods represent alternatives to the nitrogen balance technique for estimating protein requirements. Several newer methods, such as the virtual biopsy approach and ^2^H_3_-creatine dilution method could also be deployed to inform about pediatric protein requirements, although their validity and reproducibility is still under investigation. Based on the current evidence, the Dietary Reference Intakes for protein indicate that children 4–13 years and 14–18 years require 0.95 and 0.85 g·kg^−1^·day^−1^, respectively, based on the classic nitrogen balance technique. There are not enough published data to overturn these estimates; however, this is a much-needed area of research.

## 1. Introduction

The dietary protein requirements of children (persons < 19 years of age) are intended to be an estimate of the minimum continuous daily intake of “good quality” protein (e.g., an omnivorous diet) needed to prevent deficiency and ensure normal somatic growth and development [1]. The primary method used to study protein requirements is nitrogen balance; however, nitrogen balance techniques have several limitations that can lead to an overestimation of nitrogen intake and an underestimation of nitrogen excretion [2]. The net result is an overestimation of net nitrogen balance; thus, an underestimation of the requirements. Assessments of protein requirements can also be obtained by using stable isotope carbon [1] and nitrogen [3] labeled amino acids. These methods may offer an advantage over nitrogen balance by being more sensitive to rapid changes in amino acid (protein) intake and by being suitable for use in vulnerable populations (e.g., children). Compared to nitrogen balance methods, evidence from stable isotope based carbon oxidation methods have yielded higher dietary protein requirement values [4]. This may be evidence that the current requirement underestimates the minimum amount of protein needed to support childhood growth and development.

Growth may be influenced also by physical activity level. In children aged 8–15 years, moderate-to-high levels of physical activity are associated with greater lean mass and muscle strength [5,6,7]. Muscle protein breakdown and amino acid oxidation in adults increases acutely with physical activity [8]. Thus, engaging in more physical activity likely increases the need for dietary protein to replace the irreversible oxidation of essential amino acids and to support synthesis and/or maintenance of a larger muscle mass. In adults, exercise induces muscle growth and adaptation (protein deposition); this, in turn, is thought to increase the dietary protein requirements of adults [9]. In children, exercise also increases growth [5,6,7]. Since growth is one factor in the estimate for the protein requirement in children, it may be that an exercise-induced increase in protein deposition experienced by more physically active children would lead to a higher protein requirement compared to their minimally active counterparts. However, the current protein requirements do not delineate a separate requirement based on level of physical activity due to a paucity of evidence [1].

The purpose of this narrative is to review the current protein requirements in children; to review the current available evidence in children that physical activity may increase the need for dietary protein and to discuss the potential for alternative techniques to more accurately quantify the dietary protein requirements in children.

## 2. Current Protein Intake Recommendations for Children

The current protein recommendations in boys and girls < 19 years ranges from 0.85 to 1.2 g·kg^−1^·day^−1^ (Table 1) based on age group [1]. These Dietary Reference Intakes (DRI) for protein intake were established by the factorial method using data from studies relating dietary protein intake to nitrogen balance. The factorial method includes: (1) estimates of nitrogen maintenance, (2) measurements of protein deposition from body composition analysis and (3) estimates of protein utilization efficiency (rate of weight gain divided by protein quantity) [1]. The estimated average requirement (EAR) is the lowest continuing daily intake to prevent deficiency of that nutrient for 50% of the reference population; the recommended dietary allowance (RDA) is adjusted for 2 standard deviations above the average to meet the needs for 97.5% of that reference population. To accurately estimate a requirement, it is necessary to test a range of protein intakes from well below to above the expected requirement estimate. This allows for an interpolation of an expected dietary protein intake to achieve nitrogen balance. When the body is in nitrogen balance, protein breakdown presumably equals protein synthesis [10]. The assumptions are that (1) short-term nitrogen balance is reflective of long-term nitrogen balance and (2) long term nitrogen balance reflects an adequate supply of dietary protein intake to support cellular functions.

On average, children in the United States consume enough dietary protein to meet the nitrogen balance-derived requirements [11]; however, there is evidence that the true physiological requirement is even greater than nitrogen balance-derived estimates [4]. The nitrogen balance technique has several methodological issues that tend to overestimate nitrogen intake and underestimate excretion [2]. This often leads to implausibly high positive values that reflect a rate of protein deposition that does not manifest phenotypically, at least in adults [2]. Participants also require several days of adaptation to the protein intake level used for testing, another several days for measurements, full collections of urine and feces and adjustments for integumental losses [12]. This process must be repeated at a minimum of 3 protein intake levels (e.g., below, around and above the estimated requirement) to interpolate a level of protein intake where the participants are assumed to be in zero balance (nitrogen intake equaling excretion) [12,13]. In children, zero balance is considered the maintenance requirement for nitrogen. This maintenance requirement is then added to an estimate of protein deposition rate reflective of growth. Protein deposition rates were determined in children from body composition changes measured by water dilution, whole body potassium and dual energy x-ray absorptiometry [1]. Lastly, an adjustment for protein utilization efficiency is made using estimates derived from adults. The limitations of nitrogen balance have been thoroughly discussed elsewhere [2]; but the nitrogen balance technique systematically underestimates protein needs for the individual.

## 3. Physical Activity as a Modifier of Dietary Protein Requirements in Children

The current DRIs for protein do not take physical activity into consideration. In children, the protein requirement needs to simultaneously prevent a protein-related deficiency and support growth and development. In two cohort studies [5,6] and one cross-sectional study [7], greater lean mass (muscle) was observed in persons ranging in age from 8 to 21 years who engaged in moderate-to-high levels of physical activity (measured via questionnaires [5,6] and accelerometers [5,7]). Compared to relatively inactive children, greater lean mass (muscle) in active children suggests more physical activity leads to greater muscle growth. Mathematically, more physically active children would require more protein if the growth rate experienced by physically active children were greater than the growth rate included in the published requirement equations [1]. However, it is not known if consuming a protein quantity at or near the current requirement and increasing physical activity would prevent “normal” growth. Or rather, it is not known if exercise-induced growth trajectories (muscle/lean mass) are either mediated by protein intake or independent of protein intake at intakes near or above the requirement. If they are independent of protein intake, then the requirement values would not need to be adjusted for physical activity. However, if physical activity-induced growth is mediated by protein intake, then we need to determine the level of physical activity which corresponds to the appropriate protein intake level for normal muscle growth and for maximal growth. This would require us to determine an upper limit to protein and exercise beyond which no further “healthy” growth occurs. Answers to these questions would determine whether separate protein requirements are necessary for children based on physical activity level.

Muscle growth occurs when there is a positive net protein balance. At the muscle and whole-body level, a positive net protein balance occurs when protein synthesis exceeds protein breakdown. Thus, physically active children who gain more lean mass (muscle) than sedentary children must have greater net protein balance over time. This can manifest as either a more prolonged period of positive net balance or a greater difference in net balance. Regardless, greater total net balance leading to greater muscle mass and, therefore, lean body mass, would be beneficial for improving health status into later life [14]. Results from studies using the indicator amino acid oxidation (IAAO) technique demonstrate that whole-body net balance in children post-exercise increases with protein ingestion in a saturable-dose dependent manner [15,16,17]; over time, protein deposition (growth) may occur with increasing intakes of protein. However, the longitudinal effect of consuming different protein quantities on exercise-induced phenotypic changes has yet to be tested in healthy children. In adults, protein intake seems to mediate exercise-induced muscle growth [18]. The position of the Academy of Nutrition and Dietetics, Dietitians of Canada and the American College of Sports Medicine is that greater protein intakes than those outlined in the DRIs are needed to support adaptation to exercise in adults [9]. The same may be true for children as well.

While physical activity acutely increases whole-body net balance and protein turnover, muscle protein turnover is also elevated (in adults) [19]. Because amino acid recycling is not 100% efficient, higher turnover rates that occur with physical activity would suggest more dietary-derived amino acids are needed to replace the irreversible oxidation of essential amino acids [1]. There are currently no data on the rates of muscle protein turnover in children in response to an acute bout of exercise. Using an ^15^N end-product method to measure whole-body turnover, however, boys and girls (~8–10 years) beginning either a walking [20] or resistance training [21] program actually experienced a decrease in their 10-h fasted overnight protein turnover after 6 weeks. This could reflect a repartitioning of amino acids away from high turnover tissues, such as those located in the gut, towards slower turnover tissues, such as skeletal muscle [22]. The reduction in whole-body turnover could also be from a down regulation of protein turnover in response to consuming a slightly energy-restricted diet as a consequence of not increasing energy intake to compensate for the exercise [20,21]. In another comparative cross-sectional study, using the same ^15^N end-product methodology, 24 h whole-body protein turnover rates of chronically sedentary versus highly active children (soccer players and gymnasts) were not different [23,24]. This similarity may be a result of the highly active children consuming less protein than their counterparts on test days [23,24], which demonstrably results in a decrease in net protein balance [23]. Collectively, the limitations of the available evidence preclude making conclusions as to the acute or longitudinal effects of physical activity on whole-body protein turnover (Table 2). Future research should focus on following study designs that can be used to estimate the protein requirements in both minimally and highly active children: for example, testing participants at a range of protein intake levels spanning from below to above the predicted value. 

## 4. Methods to Assess Protein Metabolism in Children

In determining nutrient requirements, it is important to identify which outcomes to assess and which methods to use. In adults, a nutrient “requirement” is the minimum continuing daily intake to prevent deficiency [1]. However, in children, a nutrient should be provided in sufficient quantities to support normal growth and development [1]. The following sections will review some of the possible indicators that could be used to estimate childhood protein requirements.

### 4.1. Indicator Amino Acid Oxidation (IAAO) Method

**Figure 1:**
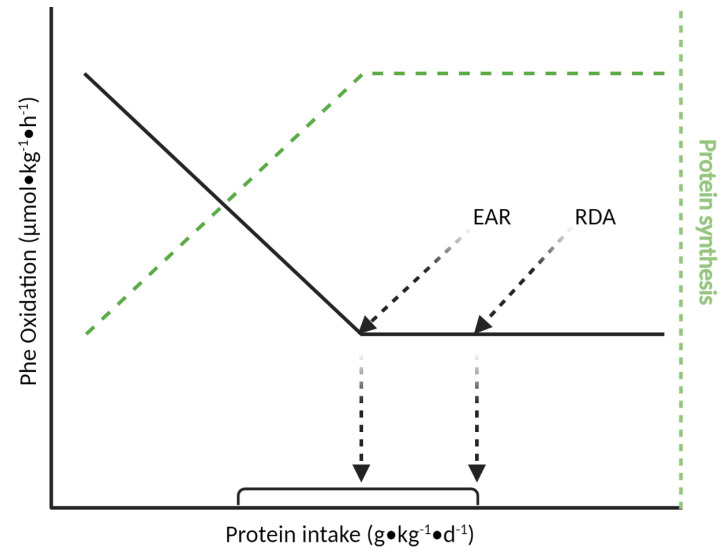
Adapted from Elango et al. [4]. As protein intake increases, so does the limiting amino acid and the oxidation of the indicator amino acid. This represents an increase in the incorporation of the indicator amino acid into newly synthesized proteins. A plateau in the oxidation of the indicator amino acid represents a maximal rate of incorporation in proteins. The breakpoint represents the estimated average requirement (EAR) for 50% of the population. The recommended dietary allowance (RDA) can be calculated as either 2 SD above the mean or the upper 95% CI. Created with BioRender.com.

The IAAO method is based on the principle that incorporation of both the indicator amino acid (e.g., L-[1-^13^C] phenylalanine (Phe)) and the other amino acids into body proteins decreases when an essential amino acid (EAA) is “limited” for protein synthesis; these “unused” amino acids are shuttled towards oxidation [25]. As intake of the limiting EAA increases with increasing grades of total dietary protein intakes, there is both greater net incorporation into body proteins and decreased oxidation of the indicator amino acid (and other amino acids). Oxidation of the indicator amino acid will no longer decrease when protein synthesis has been maximized as indicated by the breakpoint in oxidation rate [26]. The breakpoint is considered the EAR for IAAO; the RDA is considered either two standard deviations above that mean or the upper 95% confidence interval (Figure 1). Results from recent IAAO studies show the estimated protein RDA in children (6–10 y) are ~60% [13] higher, respectively, than the current requirements estimated from nitrogen balance studies (Table 1). However, no study to date has directly compared both the nitrogen balance and IAAO methods within the same participants to know how the results compare. Furthermore, no IAAO study has directly compared the requirement estimates between minimally and highly active children.

**Figure 2:**
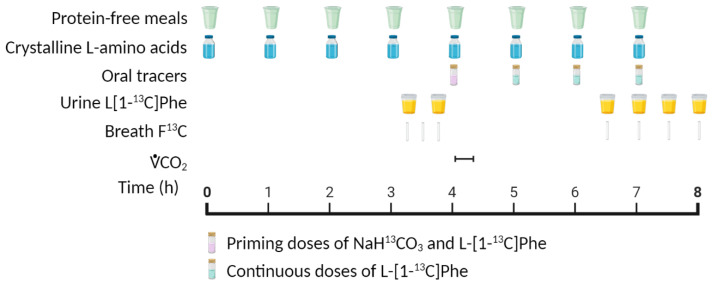
Example protocol for an indicator amino acid oxidation (IAAO) study. Created with BioRender.com.

It is worth considering several major points about the IAAO method before using it as a suitable replacement for nitrogen balance-derived requirements (Table 3). (1) A free amino acid mixture that is profiled after a high-quality protein source (typically egg) is fed to participants. Using free amino acids patterned after egg would provide a highly digestible substrate and provide an ideal amino acid profile. This would feasibly result in a lower protein requirement estimate as this would represent a “best case” scenario. In a free-living setting, dietary protein will come from plant and animal whole-food sources varying in protein quality and digestibility. (2) Measurements of oxidation occur in the anabolic fed state where protein synthesis is stimulated by the feeding events (Figure 2). Since no feeding occurs during overnight fasting, estimating the dietary protein requirement during feeding likely overestimates the daily (24 h) requirement. (3) Classic feeding studies were performed using a 12 h feeding and 12 h fasting paradigm. To achieve a steady-state during the 12 h feeding phase, 1/12 of total energy/protein is prescribed every hour. Oxidation measurements occur over the last few hours of an 8 h feeding period. Therefore, only 2/3 (8/12th) of the dietary protein intake recommended for the 24 h day (feeding and fasting) is consumed during the actual study. The metabolic responses measured at the end of the 8 h testing period (feeding only) are extrapolated to the 24 h period (feeding and fasting). (4) Protein and energy are provided as frequent small meals. This pattern of intake does not reflect actual patterns of eating behavior, which typically occur over 3–4 larger meals. Protein turnover is highly dependent on the dose of protein consumed. Thus, frequent small doses of protein would not influence protein metabolism in the same way as a larger protein dose [27]. (5) The breakpoint—the max rate of synthesis (i.e., minimum rate of indicator amino acid oxidation)—is assumed to be the minimum intake to prevent deficiency in 50% of the population (EAR). Thus, results from using the IAAO method imply that consuming less protein than what induces a maximal synthesis response is insufficient to prevent protein deficiency. Taking all these considerations together, the IAAO method likely overestimates the dietary protein requirement.

The IAAO method has several advantages: (1) it is less invasive than standard intravenous infusion protocols because urine and breath samples can be collected in place of blood; (2) it can be used to study vulnerable populations such as pregnant women or children; (3) participants only need ~2 days of adaptation to the test protein intake [28]. However, participants need to be studied at multiple protein intake levels that span the range of adequacy. This means each child must attend several in-clinic test days that last for >9 h each. While not invasive, the IAAO method can be cumbersome on participants. However, results from the IAAO method are likely less prone to error incurred by other methods used to study participants in a free-living environment.

### 4.2. ^15^N End-Product Method

**Figure 3:**
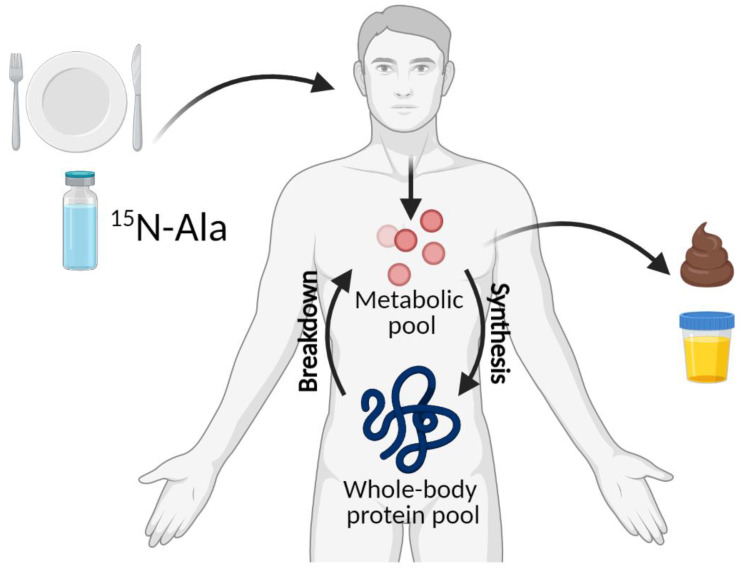
Adapted from Børsheim et al. [33] Model of whole-body protein metabolism using the N end-product method. Created with BioRender.com.

Another viable alternative for the determination of protein requirements is to use a ^15^N end-product method through the oral ingestion of a labeled nitrogen to determine nitrogen flux, or turnover, throughout the body (Figure 3). This method is based on the assumption that metabolically active nitrogen in the body is freely exchanged between nitrogen-containing tissues and the metabolic nitrogen pool [3]. Nitrogen enters the metabolic pool from both the diet and protein breakdown within the body; nitrogen leaves the pool through protein synthesis and nitrogen excretion as end-products—primarily urea or ammonia—in the urine [29]. Urea is the preferred end-product to measure because it (1) is a major source of nitrogen in the free nitrogen pool, (2) crosses cell membranes readily without creating concentration gradients, (3) is distributed in total body water, (4) represents ~90% of total nitrogen excretion and (5) is easily amenable to both clinical laboratory assessment and enrichment analysis [30]. ^15^N-Alanine is one viable labeled nitrogen carrier because it is primary involved in the inter-organ transfer of amino nitrogen [31] from the muscle, gut and kidney for urea production in the liver [32]. Oral ingestion of ^15^N-alanine (16 mg/kg [33]) and subsequent quantification of the labeled end-products (e.g., urea) has certain ecological strengths: (1) It can be used in both clinical [33,34] and field trial studies [35] to study the integrated effects of interventions (or differences in populations) in active or free-living conditions over the entire day; (2) participants can consume their normal diet and follow their regular dietary patterns; (3) measurements of whole-body protein breakdown, in addition to synthesis, can be calculated by measuring protein intake; (4) this method is less invasive than intravenous tracer infusion methods; (5) does not require the rigor or expense of pharmacy-prepared infusions. These make the end-product method a desirable method to characterize free-living changes in protein kinetics to estimate protein requirements.

**Figure 4:**
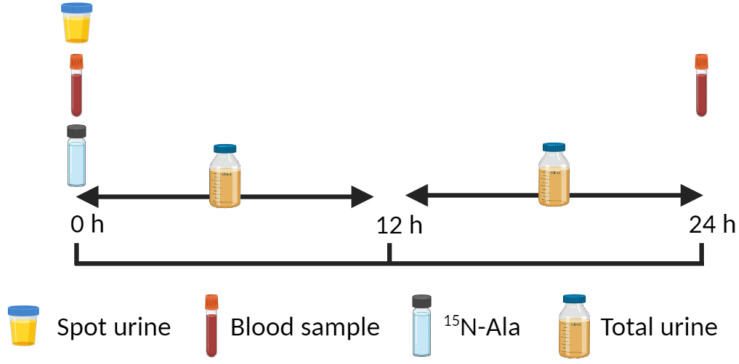
Example of a study protocol to measure whole-body protein metabolism using the N end-product method. Created with BioRender.com.

Careful characterizations of several key measures are important to the end-product method’s efficacy. First, protein intake in free-living situations must be accurately measured since protein breakdown is calculated as flux minus nitrogen intake. Nitrogen intake can be quantified by a number of methods; however, most nutritional software programs now rely upon a national database (i.e., USDA) to accurately calculate nitrogen intake through food sources. However, collecting accurate dietary data from free-living participants continues to be a challenge. Second, the change in blood urea nitrogen (BUN) pool size is important in determining interventional effects (e.g., nutritional/protein intake) on nitrogen flux. This is accomplished normally by measuring BUN at the start and end of the study period (e.g., 0 and 24 h) (Table 3; Figure 4). Third, as this is an end-product method where urinary urea nitrogen (UUN) enrichment is utilized to calculate nitrogen flux, the residual ^15^N label in BUN must be quantified at the end of the study period Fourth, nitrogen excretion must be determined accurately since protein synthesis is calculated as flux minus nitrogen excretion. Total urinary nitrogen can be determined in a pooled urine sample that encompasses the study period. A simpler method includes the determination of UUN in the pooled urine, a standard clinical laboratory measure. Since UUN entails ~90% of total nitrogen excretion [30], an estimation of total nitrogen excretion is easily derived. The actual calculation of nitrogen flux, protein synthesis, protein breakdown and net protein balance are described elsewhere [29,33,34]. If participants are studied at multiple protein intake levels, this method could provide information on a minimum (zero balance) and optimal (breakpoint in synthesis, breakdown and net balance) protein intake for children. Children could also be studied in their free-living setting to assess the effects of physical activity and fitness level on protein kinetics.

### 4.3. Emerging D_3_-Creatine Method for Assessing Protein Metabolism

There are several emerging methods to measure protein kinetics that may be suitable for estimating a protein requirement in vulnerable populations. One of the challenges for measuring protein kinetics in children is the invasiveness of traditional stable isotope infusion techniques that require blood and muscle tissue sampling [36]. However, there have been recent advances for the measurement of muscle protein synthesis using a virtual biopsy approach [36] that may make these measurements in children more feasible (Table 3). The virtual biopsy technique enables measurement of markers of protein synthesis rates in tissues by using blood samples [36]. Specifically, this approach utilizes oral intake of heavy water (^2^H_2_O) to label newly synthesized plasma proteins over periods of days, weeks, or months [36]. Combining stable isotope label incorporation with tandem mass spectrometric-based proteomics techniques to determine fractional synthesis rates (FSR) of muscle-derived proteins enables quantification of synthesis rates of hundreds of untargeted proteins after relatively low levels of in vivo ^2^H_2_O labeling [36]. The FSR of untargeted proteins is determined by quantifying the change in isotope labeling pattern induced by incorporation of deuterium into newly synthesized proteins. An untargeted approach yields a large amount of data and requires proteomics expertise. This approach would permit researchers to explore associations between plasma protein synthesis and other tissue-specific protein synthesis such as the liver; however, Shankaran et al. [37] simplified this method by using a targeted approach that is muscle specific. They found a highly significant correlation between FSR values of creatine kinase-type M and carbonic anhydrase-3 isolated from human skeletal mixed muscle and plasma [37]. This method has been validated in older men following a resistance training program during energy restriction [38]; however, more studies from different laboratories are needed to further validate the feasibility of the methodology. There are currently no data available in children.

## 5. Further Considerations

The current standards for growth are based on stature for age and weight for age growth charts put out by the Centers for Disease Control and Prevention. The data used to compile the growth charts comes from 5 national survey data sets spanning from 1963 to 1994 [39]. These charts indicate how a child’s weight and height relate to other children of the same age. For example, a child in the 50th percentile of height for age is taller than 50% of children of the same age. Both modifiable and non-modifiable factors contribute to phenotypic outcomes such as height and weight. For example, having tall parents makes one more likely to inherit genes that make a child tall. However, stunting during the developmental years can occur to children with poor nutritional status [40]. Thus, growth charts may be one tool to evaluate the efficacy of dietary interventions aimed at assessing the effect of protein quantity on changes in phenotypic outcomes that are already part of clinical practice. The follow-up question would be what level of growth is “normal?” The protein requirement is supposed to support normal growth. However, does growth continue to increase with greater protein intakes? In addition, is growth further enhanced by physical activity? Should practitioners recommend a protein intake that supports “normal” growth or “optimal” growth? Establishing what growth means is critically important in this context.

Growth is characterized by an increase in height and body mass. The composition of that body mass is primarily lean mass, as opposed to fat mass [41]. However, increasing protein intake could lead to simultaneous increases in energy intake and, thus, fat mass. The added adiposity may negatively influence the health of children, which is already a public health issue in both adults and children. The composition of that weight gain should be considered as we evaluate the influence of protein intake on growth. It may be advantageous to establish body compartment charts, such as lean mass or muscle mass for age, as indicators of growth. Currently, no standards of growth exist that utilize body compartments to assess growth status. One perspective is that if muscle growth is occurring, then all other “systems” are receiving an adequate supply of amino acids [14]. Thus, by seeking to establish lean mass (muscle) growth as a marker for the protein requirement, all other important physiological processes such as immune function, wound healing, inflammation and hormone production should be thriving. This muscle-centric view of protein intake should take into consideration that fat mass can also increase with gains in muscle [42]. Therefore, changes in the ratio of muscle to fat mass gains should be appreciated.

The current protein DRIs indicate that the requirement estimate is contingent upon consuming “good quality” protein [1]. In this instance, “good quality” is not well defined by the authors. Historically, protein quality is defined by the quantity of the essential amino acids and the digestibility of the protein source expressed as either the protein digestibility-corrected amino acid score or the digestible indispensable amino acid score [43]. Consuming a diet entirely composed of poorer quality plant sources does increase the risk for developing deficiencies and slowing growth if proper care is not taken to ensure all nine EAA are consumed from a multitude of sources [44]. Most adults and children do not eat a single protein source; rather, they consume a diet that is rich in both plant and animal sources considered relatively “poor” or “good” sources, respectively. The assumption is that consuming a balanced diet that included both plant and animal sources should be sufficient to supply the necessary amino acids to prevent deficiency and ensure proper growth and development.

## 6. Conclusions

To support growth and development, the current DRIs for dietary protein indicate that children 4–13 years and 14–18 years, require 0.95 and 0.85 g·kg^−1^·day^−1^, respectively, based on the classic nitrogen balance technique. However, results from studies designed with newer methods (IAAO) using stable isotope amino acids suggest the RDA may be closer to ~1.55 g·kg^−1^·day^−1^ for children 6–10 years. Further, physical activity may be one lifestyle factors that could increase the dietary protein requirements of children. There is currently a paucity of literature in minimally and highly active children reporting potential differences in their “true” protein requirements to ensure growth occurs normally. Research that uses a combination of stable isotope methods, such as IAAO and end-product methods (^15^N-Ala), may be one viable means to determining differences in dietary protein needs in children with different activity levels.

## Figures and Tables

**Table 1:** Current requirement estimates by age and sex for children.

Age Group	Nitrogen Balance ^1^		IAAO ^2^	
	EAR, g·kg^−1^·day^−1^	RDA, g·kg^−1^·day^−1^	EAR, g·kg^−1^·day^−1^	RDA, g·kg^−1^·day^−1^
7–12 months	1.0	1.2		
1–3 years	0.87	1.05		
4–8 years	0.76	0.95	1.30	1.55
9–13 years	0.76	0.95		
14–18 years, boys	0.73	0.85		
14–18 years, girls	0.71	0.85		

Abbreviations: EAR, estimated average requirement; IAAO, indicator amino acid oxidation; RDA, recommended dietary allowance. ^1^ From Ref. [1]. ^2^ From Ref. [13].

**Table 2:** Studies on protein intake, physical activity and whole-body protein turnover in children.

The Authors	M/F	Age, Year	Initial Training Status	Training During Measurement?	Duration	Method	Protein, g·kg^−1^⋅Period^−1^		Outcome
Bolster et al., 2001 [20]	5/2	8–10	One group	No	6 weeks. aerobic training	^15^N-Gly: 10 h overnight	>2.0		Flux, synthesis and breakdown decreased from pre to post. Net balance tended to decrease (*p* = 0.36).
Pikosky et al., 2002 [21]	7/4	7–10	One group	No	6 weeks. resistance training	^15^N-Gly: 10 h overnight	>1.5		Flux, synthesis and breakdown decreased from pre to post. Net balance tended to decrease (*p* = 0.07).
Boisseau et al., 2002 [24]	8/0,	15	Control	No	Cross-sectional	Nitrogen balance: 24 h	1.5		The control group tended to have a negative nitrogen balance compared to the athlete group, *p* > 0.05.
	11/0		Soccer players	Yes, aerobic			1.68		
Boisseau et al., 2005 [23]	0/10	7–12	Controls	No	Cross-sectional	^15^N-Gly: 24 h	1.60		Flux, synthesis and breakdown were not different between groups. Net balance was greater in the controls than in the gymnasts.
	0/10		Gymnasts	No			1.79		
Moore et al., 2014 [15]	7/6	~12	Active	Yes, aerobic	Cross-over	^15^N-Gly: 9 and 24 h	0.69	1.16	Over 9 h, net balance was greater in high protein (*p* < 0.05) than low and control, respectively, with a trend (*p* = 0.075) toward low protein being greater than control. Net balance was positive over 9 h for all conditions but only over 24 h for high protein.
							0.87	1.35	
							1.02	1.49	
Volterman et al., 2014 [16]	13/15	7–17	Active	No	Cross-over	^15^N-Gly: 16 h	0.83		Over 16 h, flux and synthesis were greater with skim milk than control and carbohydrate, respectively. Net balance was more negative with control and carbohydrate than with skim milk.
							0.82		
							1.24		
Volterman et al., 2017 [17]	7/3	9–13	Active	Yes, aerobic	Cross-sectional	^13^C-Leu: 3 h	0 g protein		Dose response increase in net protein balance.
	6/2						5 g protein		
	7/2						10 g protein		
	6/2						15 g protein		

**Table 3:** Considerations for each of the current methods to quantify protein kinetics in children.

IAAO Method	^15^N End-Product Method	D3-Creatine Method
Free amino acid mixture is used: Patterned after a high-quality protein source (typically egg). Oxidation of indicator amino acids occurs in the fed state. Small hourly meals are consumed for a steady-state: 1/12 of intake. 2/3 (8/12th) of the dietary protein intake recommended for the 24 h day (feeding and fasting) is consumed during the actual study. Protein intake during the study is known Measurements occur over the last few hours of an 8 h feeding period. The breakpoint–the max rate of synthesis (i.e., minimum rate of indicator amino acid oxidation)–is assumed to be the minimum intake to prevent deficiency in 50% of the population (EAR). Urine and breath samples can be collected in place of blood. Participants only need ~2 days of adaptation to the test protein intake. Participants need to be studied at multiple protein intake levels that span the range of adequacy. Clinical test days last ~9 h.	A single dose of isotope can be given for short measurement durations (<36 h). Measurements can take place in both a clinical and free-living setting. Participants can consume food in their normal dietary pattern (i.e., fewer, but larger meals). Less invasive than standard intravenous methods: few blood samples and urine are needed. Accurate quantification of actual protein intake can be more difficult in free-living setting. Participants need to be studied at multiple protein intake levels that span the range of adequacy. Currently, adaptation periods are not prescribed, however, 2-7 days is likely adequate. Quantification of BUN, UUN, total nitrogen, the enrichments are standardized techniques.	A dose of ^2^H_2_O is provided that can label plasma proteins over days, weeks, or months. Less invasive than traditional isotope methods: blood samples only. Participants can be studied in a free-living setting. Participants can consume food in their normal dietary pattern (i.e., fewer, but larger meals). Accurate quantification of actual protein intake can be more difficult in free-living setting. Target metabolomics approaches could make the method easier.
